# When Evidence Is Scant, Mathematical Modeling Offers a Roadmap for Discovery

**DOI:** 10.1371/journal.pbio.0040323

**Published:** 2006-09-12

**Authors:** Liza Gross

Scientists often talk about the value of intuition in guiding them toward the big questions. But when it comes to figuring out how all the pieces of a complex system fit together—with hordes of uniquely behaving components interacting in nonlinear pathways—systems biologists prefer to rely on computers. Theoretical analysis has increasingly been applied to understanding protein, gene, and biochemical networks in cells. Most cell network models either generate dynamic, quantitative descriptions (specifying the timing and kinetics of interactions) of well-defined pathways but use relatively few components, or present static maps of protein–DNA or protein–protein interactions that cover the entire genome but lack quantitative and dynamic information.

In a new study, Song Li, Sarah M. Assmann, and Réka Albert create a dynamic model, based on a wide range of experimental observations that describes a complex signaling network in plants that is initiated by a plant hormone called abscisic acid (ABA). They circumvent a lack of quantitative data, especially concerning interaction strengths and reaction rates, of components in ABA signaling by using a computational technique that implicitly incorporates a range of possible quantitative parameters. Their model describes the regulation of over 40 network components, demonstrates the network’s response to a range of perturbations through simulations, and makes novel, testable predictions about the sensitivity of the signaling pathway.

Plant growth and survival depend on the regulation of stomatal pores, which allow both carbon dioxide uptake for photosynthesis and water release through evapo-transpiration. Signaling pathways triggered by ABA (a stress hormone secreted by the roots and synthesized by guard cells surrounding the pores), inhibit stomatal opening and promote closure, allowing the plant to conserve water during drought. ABA signaling triggers changes in cytosolic calcium through intermediate messengers that regulate the release of calcium from internal stores or the import of extracellular calcium; it also triggers the increase of cytosolic pH, and modulates a number of enzymes and cellular metabolites. As a result, membrane-localized ion channels open, releasing potassium ions and the negatively charged chloride and malate ions, leading to stomatal closure.

To shed light on ABA signaling dynamics, Li et al. synthesized published experimental data, mostly from *Arabidopsis thaliana* studies, about the components and processes of ABA signaling into a theoretical network. Experimental evidence described either direct interactions between components (from biochemical data on enzymatic activity or protein–protein interactions) or inferences about pathway activity (from genetic mutations or pharmacological interventions). These annotated components formed nodes (and intermediate nodes, representing unknown mediators) in the network, and the annotated processes provided the basis for writing algorithms describing possible interactions between the components and constructing the paths of the network.

**Figure:**
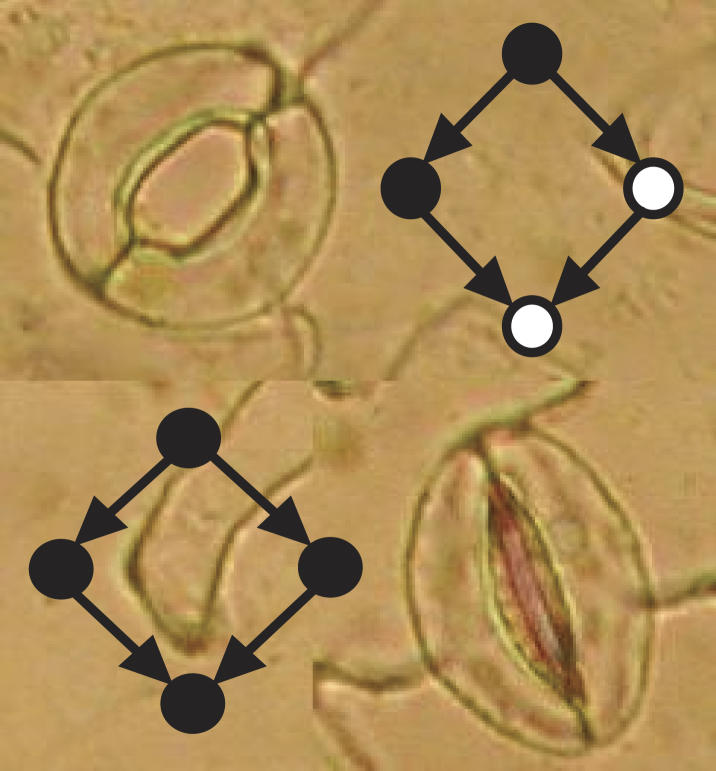
The authors model guard cell opening (upper panel) and closing (lower panel) in response to the plant hormone abscisic acid. (Image: Song Li)

Li et al. determined all the paths that nodes participated in and simulated experimental perturbations to individual nodes to predict how the network structure responds to such disturbances. They found several independent paths between the ABA input and the stomatal closure output. For example, the path involving pH-induced anion efflux does not overlap with paths regulating calcium levels, which can in turn be elevated through several independent paths. This independence, the researchers argue, indicates a “remarkable topological resilience” in which functionally redundant paths maintain ABA sensitivity in the face of disruptions in other pathways.

The path analysis describes routes from input to output, but can’t reveal synergism between nonoverlapping paths. To do this, Li et al. used a dynamic modeling technique based on binary (active/on or inactive/off) assumptions about the state of interacting nodes to predict the probability of stomatal closure. Stomata don’t usually open or shut completely, however, but close to varying degrees. The researchers captured this individual variability in the model by measuring stomatal apertures in *Arabidopsis* plants in the presence or absence of ABA application. These experiments provided population-level data to set a threshold between the open and closed state. With scant data on interaction times, component decay rates and initial states, they randomly assigned these values to cover the range of possibilities in over 10,000 simulations. They found that ABA induces complete closure in eight time steps; without ABA, the probability of closure is zero by the sixth step.

Systematically perturbing this dynamic model system—simulating the effects of knocking out a gene or pharmacologically inhibiting a protein’s activity—identified three single disruptions that made the system insensitive to ABA: loss of membrane depolarizability (which would prevent potassium channels from opening), disruption of anion efflux, and loss of actin cytoskeleton reorganization. Simulating two or three perturbations at a time showed that most of these multiple disruptions resulted in reduced (but not completely lost) ABA sensitivity. Simulated perturbations also predicted the integral contribution of cytosolic pH increase to ABA signaling—which Li et al. confirmed by showing that experimentally clamping cellular pH levels blocks stomatal closure. These results suggest that experimentalists would do well to further explore the role of cytosolic pH in ABA signaling.

While the researchers acknowledge that their network reconstruction is incomplete, it will easily incorporate new nodes as new signaling components are discovered and become more robust as quantitative experimental data emerge. This study shows how mathematical modeling and theory can synthesize a body of incomplete information on signal transduction to make predictions about the relative importance and behavior of network components. And with these predictions, experimentalists can focus their investigations on the most promising avenues of inquiry to reveal fundamental insights into the dynamics of complex biological systems.

